# Functional Analysis of MAX2 in Phototropins-Mediated Cotyledon Flattening in *Arabidopsis*

**DOI:** 10.3389/fpls.2018.01507

**Published:** 2018-10-17

**Authors:** Qing-Ping Zhao, Xiao-Nan Wang, Nan-Nan Li, Zi-Yi Zhu, Shi-Chao Mu, Xiang Zhao, Xiao Zhang

**Affiliations:** Institute of Plant Stress Biology, State Key Laboratory of Cotton Biology, School of Life Sciences, Henan University, Kaifeng, China

**Keywords:** cotyledon flattening, hypocotyl elongation, MAX2, phototropin, phototropism

## Abstract

Phototropins (phot1 and phot2) are blue-light receptors that control cotyledon flattening and positioning under strong light; however, their functional redundancy restricts our understanding of the specific roles of phot2. To identify the factors responsible for phot2-dependent cotyledon flattening and growth, we screened for light-insensitive mutants among mutagenized *phot1* mutants in *Arabidopsis thaliana*. The double mutant *phot1* *lea1* (*leaf expansion associated 1*), which is defective in cotyledon flattening and positioning but not the phototropic response was selected. This mutant phenotype could be alleviated by constitutively expressing *MORE AXILLARY GROWTH 2* (*MAX2*), indicating that *LEA1* was allelic to *MAX2*. The *max2* mutants (*max2-2* and *max2-3*) are defective in cotyledon flattening, which is similar to that of the *phot1 phot2* mutants. Moreover, the amounts of MAX2 transcripts are inhibited in leaves of *phot1* mutant. However, the additional disruption of *PHOT1* gene in *max2-2* or *max2-3* did not affect their phenotype, including MAX2-mediated inhibition of hypocotyl elongation. By contrast, phototropins-mediated hypocotyl phototropism was not regulated by MAX2. Together, these results suggest that cotyledon flattening was mediated by both phototropins and MAX2 signaling, but the relationship between two pathways need further study.

## Introduction

Light provides not only energy but also several environmental signals important for plant growth. Plants can sense changes in light intensity, quality, and direction and alter their growth in response, including regulating parameters of the leaf morphogenesis and movement, to optimize light capture and increase photosynthetic productivity (Fankhauser and Christie, 2015). Blue light affects the formation and distribution of new leaves, regulating their thickness and area by increasing the thickness of the epidermis and mesophyll cell growth (Moreira da Silva and Debergh, 1997). The phototropins (phot1 and phot2) are blue-light receptors that detect blue light and, in response, regulate many physiological activities, such as hypocotyl phototropism (Sakai et al., 2001), stomatal opening (Kinoshita et al., 2001), chloroplast relocation (Kagawa et al., 2001), and leaf positioning and flattening (de Carbonnel et al., 2010).

Leaves are the power houses of plants, providing energy for all organs through the process of photosynthesis (Van Dingenen et al., 2016). Leaf flattening and positioning maximizes light capture and increases photosynthetic productivity (Walters, 2005; Jiao et al., 2007). The cotyledonary petiole angle, the angle between the cotyledonary petiole and the horizontal plane, and the cotyledon angle between the petiole and cotyledonary blade (Harada et al., 2013) can be determined in different growing conditions to better elucidate the regulation of leaf positioning and flattening. Previously, these processes were found to be mainly regulated by phot1 under weak white light, but by both phot1 and phot2 under moderate and strong levels of white light (Harada et al., 2013). In addition to phototropin-mediated phototropism, NPH3 (NON-PHOTOTROPIC HYPOCOTYL 3) (Inoue et al., 2008), RPT2 (ROOT PHOTOTROPISM 2) (Harada et al., 2013), and the PKSs (PHYTOCHROME KINASE SUBSTRATES) (de Carbonnel et al., 2010) are also involved in leaf positioning and flattening. Plant hormones, especially auxin, are believed to affect leaf flattening, because the distribution of auxin is altered in developing leaves that go on to have different leaf flattening phenotypes (Lumba and McCourt, 2005). CPT1 (coleoptile phototropism1) and the PKS (PHYTOCHROME KINASE SUBSTRATES) regulate the asymmetric distribution of auxin in the coleoptiles (Haga et al., 2005) and leaves (de Carbonnel et al., 2010), while phot1 likely interacts with NPH3/RPT2 family proteins via the PKSs to regulate the activity and position of the auxin efflux carrier PIN (PIN-FORMED) 1 and the auxin influx transporter AUX (AUXIN) 1 (Blakeslee et al., 2004; Stone et al., 2008). The phot1-PKS-NPH3 complex therefore plays an important role in both phototropism (Fankhauser and Christie, 2015) and leaf positioning and flattening; however, the mechanism by which this protein complex functions largely unknown, although it may involve EHB1 (ENHANCED HYPOCOTYL BENDING 1) (Knauer et al., 2011). Phot2 was reported to be involved in the control of leaf flattening and positioning independently of phot1 and NPH3 (Inoue et al., 2008). These results demonstrated that numerous factors and pathways participate in the regulation of leaf positioning and flattening.

Although phot2 was reported to participate in controlling leaf flattening and positioning, the functional redundancy of phot1 and phot2 has limited previous attempts to investigate the specific role of phot2 in these mechanisms. To identify downstream factors of phot2 signaling while avoiding interference by phot1, the *phot1* mutant was further mutagenized and used for screening defective leaf positioning and flattening, resulting in the identification of the *phot1* *lea1* (*leaf expansion associated 1*) mutant. This mutant contained polymorphisms in both *MORE AXILLARY GROWTH 2* (*MAX2*) and *MAX3*; therefore, we investigated the functions of MAX2 and MAX3 in leaf positioning and flattening, and explored the relationships between the phototropins and the MAX proteins.

## Results

### Isolation and Characterization of the Leaf Positioning and Flattening Defect Mutant *phot1* *lea1*

**FIGURE 1:**
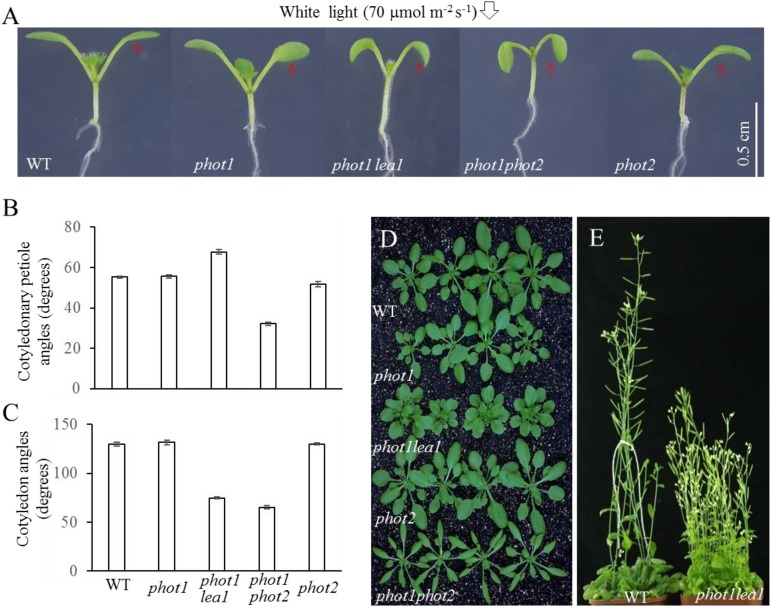
Phenotype analysis of *phot1 lea1* and related mutants. **(A)** Cotyledon flattening and positioning of wild-type (WT), *phot1*, *phot1 lea1*, *phot1 phot2*, and *phot2* plants grown for 7 days under white light. **(B)** Cotyledonary petiole angle of seedlings represented in panel **(A)**. **(C)** Cotyledon angle of seedlings represented in panel **(A)**. Each bar represents an average of three experiments (15–20 measurements per experiment) ± SE. **(D)** True leaves of WT, *phot1*, *phot1 lea1*, *phot1 phot2*, and *phot2* plants grown under white light for 1 month. **(E)** Phenotype of WT and *lea1* at maturity.

Under 70 μmol m^−2^ s^−1^ white light, the cotyledonary petioles of the wild type (WT), *phot1*, and *phot2* plants grew obliquely upward, resulting in flat and almost horizontal cotyledons (**Figure 1A**) with similar cotyledonary petiole angles and cotyledon angles (**Figures 1B,C**). The cotyledonary petiole of the *phot1 phot2* double mutant emerged more or less horizontally, resulting in an almost vertical cotyledon (**Figures 1A,C**). This suggests that *PHOT1* and *PHOT2* contribute redundantly to cotyledon positioning and flattening in *Arabidopsis thaliana* as reported previously (Harada et al., 2013).

In order to identify factors downstream of phot2 signaling and avoid the interference of phot1, the *phot1* mutant was mutated using 0.3% (w/v) EMS (ethylmethane sulfonate). We identified a suspected heritable mutant with defects in leaf flattening and positioning, which was subsequently named *phot1* *lea1* (**Figure 1A**). In comparison with the WT, *phot1*, and *phot2* plants, *phot1* *lea1* exhibited a greater cotyledonary petiole angle and a smaller cotyledon angle (**Figures 1A,C**). Although the cotyledon angle in *phot1* *lea1* (65.3°) was similar to *phot1 phot2* (74.8°), the cotyledonary petiole angle of *phot1* *lea1* (67.7°) was much larger than that of *phot1 phot2* (32.2°). Both *phot1* *lea1* and *phot1 phot2* had downward-pointing (epinastic) cotyledon blades (**Figure 1A**), although their true leaves differed; the *phot1* *lea1* had shortened leaf petioles and broader, more highly lobed leaves, while *phot1 phot2* double mutant leaves were elongated with curled margins (**Figures 1D,E**). In addition, *phot1* *lea1* formed more rosette leaves in the vegetative stage (**Figure 1D**) and had increased shoot branching in the flowering stage (**Figure 1E**). These results indicated that LEA1 played an important role in leaf positioning and flattening.

The F_1_ progeny from crosses between Col-0 and *phot1* *lea1* had the WT phenotype, and the subsequent F_2_ individuals had a segregating cotyledon phenotype. The phenotypes of the F_2_ population segregated at a ratio of three WT to one mutant (721:226, χ^2^ = 0.12), suggesting that the phenotype of *phot1* *lea1* resulted from a single recessive mutation (**Supplementary Table 1**). Crosses between *phot1* *lea1* and *Landsberg erecta* resulted in the same conclusion (**Supplementary Table 1**) and provided material for further map-based cloning. These results indicated that the single mutation of LEA1 caused downward curled cotyledon blades, and this phenotype is independent of *phot1* background.

### Both MAX2 and MAX3 Have Base-Substitution Mutations in the *phot1* *lea1* Mutant

The *lea1* mutation was identified using map-based cloning and sequencing of the segregating F_2_ population derived from the cross between *lea1* and *Landsberg erecta*. DNA extracted from more than 100 selected seedlings was used to analyze their recombination frequency at nine different molecular makers. The statistical analysis results (**Supplementary Table 2**) showed that the recombination ratio was lowest at T16B24 (12.93%), indicating that the *LEA1* gene was located closest to this marker on chromosome 2. To further narrow the interval, fine mapping was performed using another seven markers located up- and downstream of the T16B24 marker. The recombination frequency was lowest at F14N22 (0.56%) (**Supplementary Table 3**). The full length of the F14N22 BAC marker sequence is approximately 96.69 kb, including a 19.39-kb overlap with F7D19 on one side and a 7.13-kb overlap with MHK10 on the other. There are 19 candidate genes located on the F14N22 BAC, including the overlap regions (**Supplementary Table 4**), and DNA sequencing of these genes indicated the presence of a C-to-T substitution mutation in *AT2G42620* (**Supplementary Figure 1A**) in the *phot1* *lea1* mutant, which caused a premature stop codon (**Supplementary Figure 1B**).

*AT2G42620* encodes MAX2, a member of the MAX family protein. The four *MAX* genes have previously been found to have redundant functions in axillary branching (Bennett et al., 2006), and strigolactone biosynthesis and signaling (Jia et al., 2014). To rule out the influence of functionally redundant MAX proteins, all *MAX* genes were amplified and sequenced in *lea1*. In addition to *MAX2*, *MAX3* (*AT2G44990*) also had a C-to-T substitution at position 1270 in *lea1* (**Supplementary Figure 1C**), which caused the premature termination of transcription (**Supplementary Figure 1D**). These results indicated that the defect of leaf positioning and flattening in *lea1* is likely mediated by both *MAX2* and *MAX3*.

### The *max2* Mutant Showed Defects in Hypocotyl Elongation, and Leaf Positioning and Flattening

**FIGURE 2:**
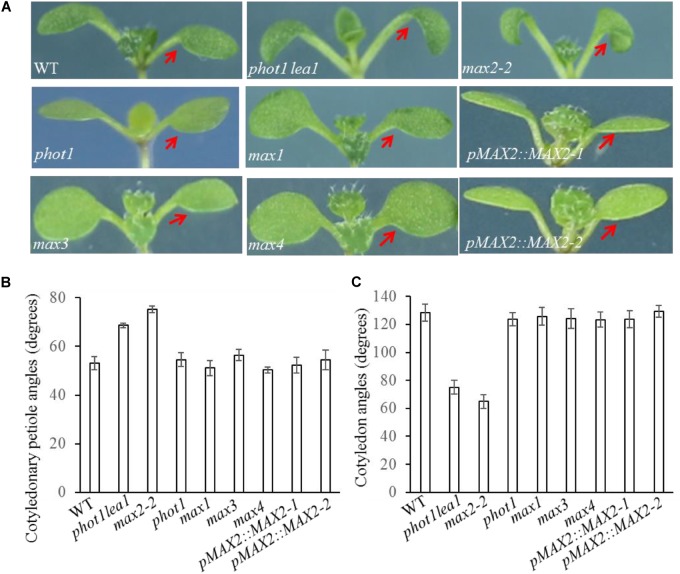
Cotyledon flattening and positioning in the WT, and the *phot1 lea1* and *max* mutants. **(A)** Cotyledon flattening and positioning of the WT, *phot1 lea1*, *max* mutants, and transgenic plants (*phot1 lea1* mutants with complemented *MAX2* expression driven by the native promoter). Red arrows indicate the cotyledons. **(B)** Cotyledonary petiole angles of seedlings represented in panel **(A)**. **(C)** Cotyledon angles of seedlings represented in panel **(A)**. Each bar represents an average of three experiments (15–20 measurements per experiment) ± SE.

**FIGURE 3:**
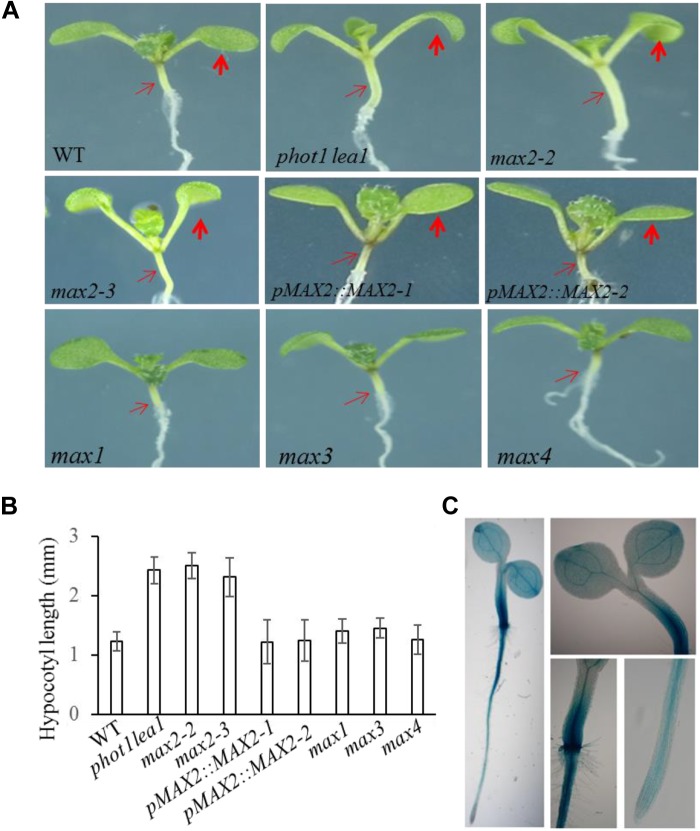
Hypocotyl elongation of WT, *phot1 lea1*, and the *max* mutants. **(A)** Hypocotyl elongation of WT, *phot1 lea1*, *max* mutants, and transgenic plants (*phot1 lea1* plants with complemented *MAX2* expression driven by the native promoter). The bold red arrows indicate the cotyledons, while the fine red arrows indicate the hypocotyls. **(B)** Hypocotyl length of seedlings represented in panel **(A)**. Each bar represents an average of three experiments (15–20 measurements in each experiment) ± SE. **(C)** Tissue localization of *MAX2* expression. GUS staining of transgenic plants revealed the *MAX2* expression in the roots, leaves, and hypocotyl.

As previously reported, all *max* mutants produced more rosette leaves (**Supplementary Figure 2B**), had higher levels of axillary branching (**Supplementary Figure 2A**), and formed shorter primary inflorescences (**Supplementary Figure 2A**) than the WT. The functional redundancy of the MAXs in shoot branching and the two base substitution mutations found in *phot1* *lea1* suggested that cotyledon positioning and flattening may also be redundantly regulated by MAX2 and MAX3; however, only the *max2* mutants had impaired cotyledon positioning and flattening similar to *phot1* *lea1* (**Figure 2**). The *max2-2* and *max2-3* allelic mutants had larger cotyledonary petiole angles and smaller cotyledon angles than the WT, and downward curled cotyledon blades similar to *phot1 phot2* (**Figures 2B,C**). In addition, *phot1* *lea1* and *max2* have other phenotypes not observed in the WT or the other *max* mutants, such as longer hypocotyls (**Figures 3A,B**) and flat siliques (**Supplementary Figure 2C**).

In order to further demonstrate that the phenotypes in *phot1* *lea1* are caused by the disruption of *MAX2*, we complemented the *phot1* *lea1* mutant using the *MAX2* gDNA sequence under the control of the native *MAX2* promoter (a 2.0-kb fragment upstream of the start of translation). Two independent transgenic lines (*pMAX2*::*MAX2-1* and *pMAX2*::*MAX2-2*) rescued the *phot1* *lea1* leaf flattening and positioning phenotype and the hypocotyl elongation defect in T_3_ homozygous plants (**Figures 2**, **3**). The transgenic *pMAX2*::*MAX2-1* and *pMAX2*::*MAX2-2* plants in the *phot1* *lea1* mutant background that the expression of *MAX2* gene has been restored(**Supplementary Figure 3**), had almost horizontal and flat cotyledon blades and shorter hypocotyls, as observed in the WT (**Figures 2**, **3**). The other phenotypes, including the shape of the silique, the number of rosette leaves, and the axillary branching, were also restored (data not shown). These results indicated that MAX2, but not MAX3, regulates hypocotyl length, silique morphology, and leaf positioning and flattening, despite the mutation of both *MAX2* and *MAX3* in *lea1*.

The *MAX2* expression pattern was examined in transgenic plants expressing *β-glucuronidase* (*GUS*) under the control of the *MAX2* promoter. GUS staining revealed that *MAX2* was ubiquitously expressed in all vegetative tissues during the seedling stage (**Figure 3C**), as previously reported (Shen et al., 2007). The joint between the hypocotyl and the root had the strongest GUS activity, while the leaves and hypocotyls had higher levels of *MAX2* expression than the root (**Figure 3C**). These results support the developmental phenotype that MAX2 is mainly involved in leaf flattening and positioning, hypocotyl elongation, and shoot branching regulation.

### The *phot1 max2-2* and *phot1 max2-3* Double Mutants Exhibited Defects in Cotyledon Flattening

**FIGURE 4:**
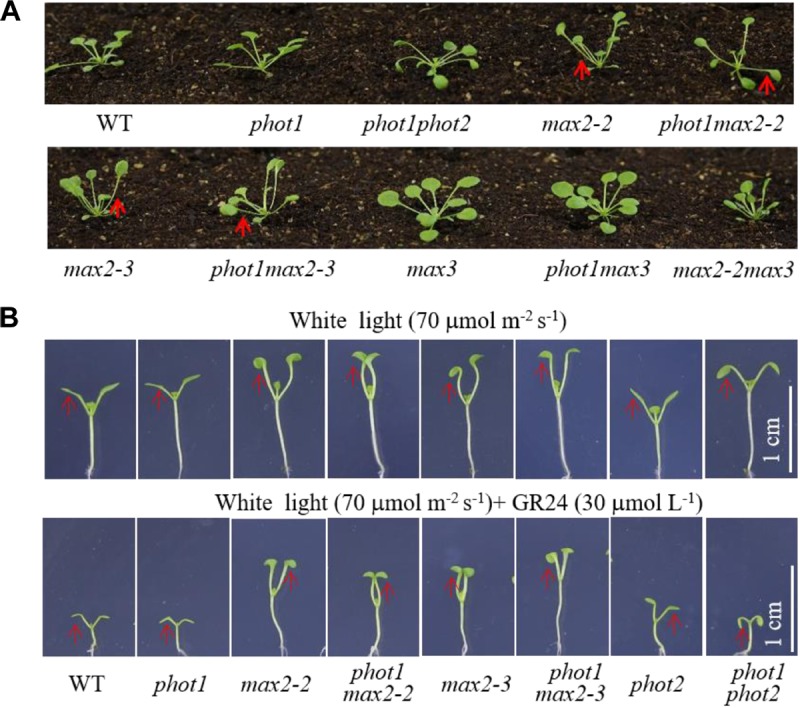
Leaf flattening and positioning and hypocotyl elongation of WT and mutant plants. **(A)** Leaf flattening and positioning in the WT and mutants grown under 70 μmol m^−2^ s^−1^ white light for about 4 weeks. Red arrows indicate the different leaf positioning phenotypes between the single and double mutants. **(B)** Hypocotyl elongation of 7-day-old WT and mutant plants under 70 μmol m^−2^ s^−1^ white light, with or without 30 μmol L^−1^ GR24. Scale bars represent 1 cm.

Under weak white light (≤10 μmol m^−2^ s^−1^), phot1 is known to control leaf flattening and positioning alone, while in increasing light intensities (≥25 μmol m^−2^ s^−1^), both phot1 and phot2 regulate the responses (Harada et al., 2013). We have demonstrated that MAX2 also plays an important role in leaf flattening and positioning; therefore, to investigate the relationship between the two pathways, we characterized the *phot1 max2-2* and *phot1 max2-3* double mutants. With respect to leaf phenotype, *phot1 max2-2* and *phot1 max2-3* exhibited defects in leaf flattening and positioning similar to those of *max2-2* and *max2-3* (**Figure 4A**), and all four of these mutants had the same cotyledon flattening phenotype as *phot1 phot2* (**Figure 4B**). Mutating *PHOT1* in the *max3* mutant background had no effect on leaf flattening and positioning, whereas the *max2 max3* double mutant had leaf flattening and positioning defects similar to *max2-2* (**Figure 4A**). These results indicated that both phototropins and MAX2 function in cotyledon flattening regulation, but their relationship need further study.

### Both phot1 and phot2 Had Little Effect on MAX2-Regulated Hypocotyl Inhibition

The *phot1 max2-2* and *phot1 max2-3* double mutants produced long hypocotyls similar to those of the *max2-2* and *max2-3* single mutants, all of which were longer than those of the WT, *phot1*, *phot2*, and *phot1 phot2* plants under 70 μmol m^−2^ s^−1^ white light (**Figure 4B**). When treated with 30 μmol L^−1^ GR24, hypocotyl elongation was reduced in all genotypes investigated, although the reductions in *max2-2*, *max2-3*, *phot1 max2-2*, and *phot1 max2-3* were smaller than in WT, *phot1*, *phot2*, or *phot1 phot2* (**Figure 4B**). These results indicated that MAX2 regulated the strigolactone-inhibited elongation of the hypocotyls, and that this was not affected by the disruption of *PHOT1*.

**FIGURE 5:**
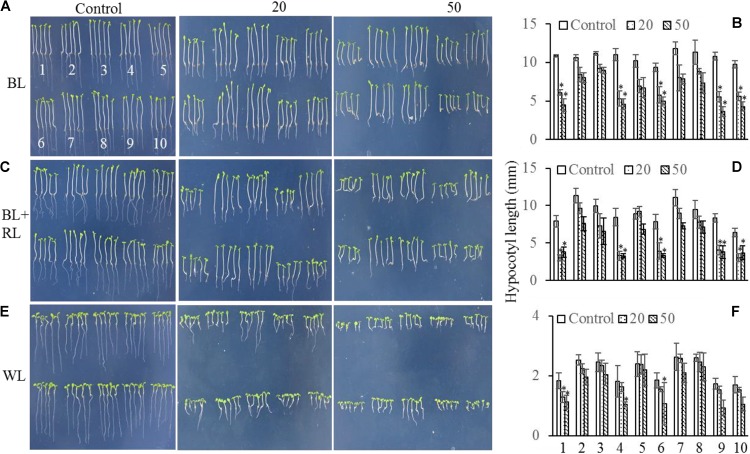
Hypocotyl elongation of WT and mutant plants under various light conditions and treated with 0, 20, or 50 μmol L^−1^ GR24. **(A,C,E)** Phenotypes of 5-day-old WT and mutant seedlings grown on MS plates with 0, 20, or 50 μmol L^−1^ GR24 under **(A)** 10 μmol m^−2^ s^−1^ blue light (BL), (C) 10 μmol m^−2^ s^−1^ blue light plus 10 μmol m^−2^ s^−1^ red light (BL + RL), or **(E)** 70 μmol m^−2^ s^−1^ white light (WL). **(B,D,F)** Hypocotyl lengths of seedlings in panels (A,C,E), respectively. Each bar represents an average of three experiments (15–20 measurements per experiment) ± SD. ^∗^*P* < 0.05. The seedling identifiers are as follows: 1, WT; 2, *max2-2*; 3, *max2-3*; 4, *max3*; 5, *max2-3 max3*; 6, *phot1*; 7, *phot1 max2-2*; 8, *phot1 max2-3*; 9, *phot1 max3*; 10, *phot1 phot2.*

The mechanism controlling the MAX2-dependent inhibition of hypocotyl growth only functioned at low concentrations of GR24 (Jia et al., 2014), suggesting the presence of a MAX2-independent pathway regulating hypocotyl elongation at high concentrations of GR24. *MAX3* encodes a carotenoid cleavage dioxygenase (Sorefan et al., 2003; Booker et al., 2004) that promotes strigolactone biosynthesis. Phot1 has also been reported to inhibit hypocotyl elongation (Folta et al., 2003); therefore, we used different concentrations of GR24 to analyze the inhibition of hypocotyl elongation in WT, *max2-2*, *max2-3*, *max3*, *max2-2 max3*, *phot1*, *phot1 max2-2*, *phot1 max2-3*, *phot1 max3*, and *phot1 phot2* plants. As previously reported, hypocotyl elongation was inhibited in all seedlings following a treatment with 50 μmol L^−1^ GR24 (Jia et al., 2014) in combination with light irradiation (**Figure 5**); however, when the concentration of GR24 was decreased to 20 μmol L^−1^, the inhibition of hypocotyl elongation became more complex. Under white light or a combined red and blue light, the hypocotyl elongation was reduced in all seedlings except for *max2-3 max3*, whose length showed almost no difference (**Figures 5C–F**). Overall, the decrease in hypocotyl elongation under blue light was greater than under the blue- and red-light combination. The *max2-2*, *max2-3*, *phot1* *max2-2*, and *phot1 max2-3* plants had smaller decreases in hypocotyl length under both conditions (**Figures 5A–D**). Under white light, the pattern of hypocotyl length in seedlings of the various genotypes was the same as under blue light, except that the extent of the decrease was reduced (**Figures 5E,F**). Taken together, these results show that MAX2 participates in strigolactone-mediated hypocotyl elongation, as previously reported (Jia et al., 2014), but that the phototropins and MAX3 have no effect on this response under the conditions observed here.

### Neither MAX2 nor MAX3 Regulate Hypocotyl Phototropism

Beyond leaf flattening and positioning, phot1 and phot2 redundantly regulate high-intensity blue light (HBL)-induced hypocotyl phototropism (Sakai et al., 2001; Zhao et al., 2013), while MAX2 is known to participate in the regulation of hypocotyl elongation (Stirnberg et al., 2002). Hypocotyl elongation and phototropism are distinct physiological processes, but ultimately both depend on changes in cell elongation (Liscum et al., 1992); therefore, hypocotyl phototropism was analyzed in WT, *phot1*, *max2-2*, *phot1 max2-2*, *max2-3*, *phot1 max2-3*, *max3*, *phot1 max3*, *max2-2 max3*, and *phot1 phot2* plants.

**FIGURE 6:**
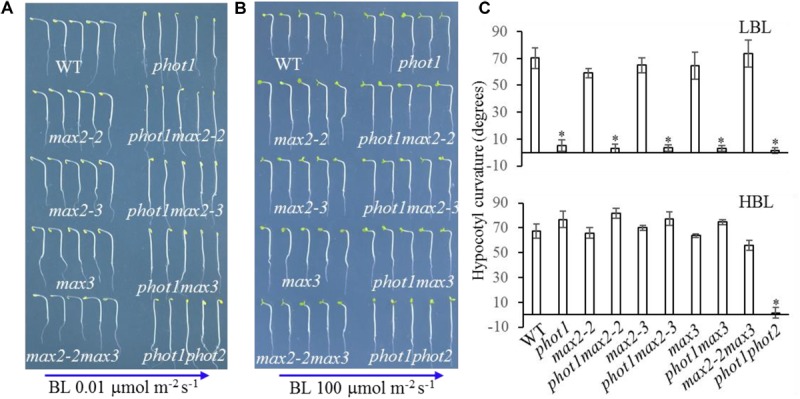
Hypocotyl phototropism of WT and mutant seedlings. **(A,B)** Three-day-old etiolated WT and mutant seedlings were irradiated with blue light at **(A)** 0.01 μmol m^−2^ s^−1^ or **(B)** 100 μmol m^−2^ s^−1^ for 12 h. **(C)** Hypocotyl curvatures of seedlings in panels **(A,B)**. Each bar represents an average of three experiments (15–20 measurements per experiment) ± SD. ^∗^*P* < 0.05.

Under high levels (100 μmol m^−2^ s^−1^) of blue light, all genotypes displayed normal phototropism, except for *phot1 phot2* (**Figures 6B,C**), demonstrating that MAX2 has no effect on HBL-induced phototropism. Under low levels (0.01 μmol m^−2^ s^−1^) of blue light, the phototropic responses of *max2-2*, *max2-3*, *max3*, and *max2-2 max3* resembled that of the WT (**Figures 6A,C**). When *PHOT1* was disrupted in the *max2-2* and *max2-3* mutant backgrounds, the resulting double mutants showed impaired phototropism, similar to *phot1* (**Figures 6A,C**). These results were consistent with the previous report demonstrating that phot1 regulates low-blue-light-induced phototropism (Briggs et al., 2001), and show that MAX2 is not required for phot1-dependent phototropism.

**FIGURE 7:**
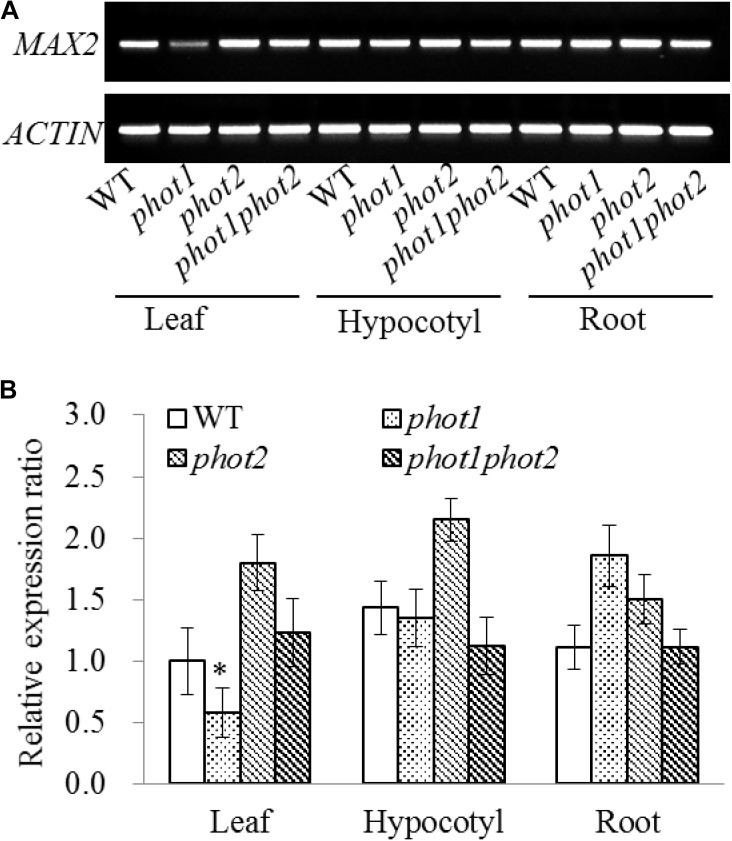
Gene expression of *MAX2* in the WT and *phot* mutants by RT-PCR and qRT-PCR. **(A)** RT-PCR analysis of *MAX2* gene expression levels in the leaves, hypocotyls, and roots of 7-day-old WT, *phot1*, *phot2*, and *phot1 phot2* plants. **(B)** Expression of *MAX2* in the leaves, hypocotyls, and roots of 7-day-old WT, *phot1*, *phot2*, and *phot1 phot2* plants, determined using quantitative real-time PCR (qRT-PCR). Values are the means of three biological repeats ± SD. ^∗^*P* < 0.05.

Seven-day-old WT, *phot1*, *phot2*, and *phot1 phot2* seedlings were dissected into their leaf, hypocotyl, and root tissues to investigate the expression of *MAX2*. The results of the RT-PCR and RT-qPCR indicated that *MAX2* was almost equally expressed in all three tissues in these plants, and the mutation of *PHOT1* gene inhibited the expression of *MAX2* in leaves (**Figure 7**).

## Discussion

### MAX2 Is Necessary for Leaf Flattening and Positioning

Genetic studies have suggested that the phototropins (phot1 and phot2) function redundantly to control leaf flattening and positioning under strong light in *Arabidopsis thaliana* (Harada et al., 2013). Phot1 is believed to inhibit the phot2 signaling pathway by influencing the downstream components of phot2 (Harada et al., 2013); however, the complex functions of phot1 and phot2 in leaf flattening and positioning have restricted our understanding of the contribution made by phot2. Here, we attempted to identify new signaling components acting downstream of phot2 by screening an EMS-mutagenized population in the *phot1* mutant background to avoid interference from phot1. This approach enabled the identification of the *lea1* mutant, which showed defects in leaf flattening and positioning, and had hook, epinastic cotyledon blades similar to the *phot1 phot2* double mutant (**Figure 1**). Subsequently, we cloned the mutated gene in *phot1* *lea1* and demonstrated that *LEA1* was allelic to *MAX2*. Consistently, two null mutants of *MAX2* (*max2-2* and *max2-3*) showed the same curled cotyledon blade phenotype as *phot1* *lea1* and *phot1 phot2*.

MAX2 is one of four known members of the MAX family. *MAX3* and *MAX4* encode divergent carotenoid cleavage dioxygenases (Sorefan et al., 2003; Booker et al., 2004) which promote strigolactone biosynthesis from a carotenoid-derived substance (Jia et al., 2014). *MAX1* encodes a cytochrome P450 family member that acts downstream of MAX3 and MAX4 (Bennett et al., 2006), while MAX2 contains an F-box motif, a repeat sequence segment of 18 leucine residues, and a zinc-finger domain (Woo et al., 2001). The F-box protein encoded by *MAX2* is a part of the SCF complex that directs the E3-mediated protein hydrolysis process (Stirnberg et al., 2007). All *MAX* genes redundantly regulate axillary branching (Bennett et al., 2006), and strigolactone biosynthesis and signaling (Jia et al., 2014). Consistently, here we found that all *max* mutants have increased numbers of rosette leaves and higher levels of axillary branching (**Supplementary Figure 2**) but only *max2* seedlings have elongated hypocotyl, elevated cotyledonary petioles and downward-curled cotyledons (**Figure 2**). These results indicated that MAX2 was necessary for cotyledon flattening but this signaling was not conventional MAX3 and MAX4-dependent metabolism of strigolactone. Karrikins are other butenolides plant growth regulators, which is structurally similar but physiologically distinct from strigolactones (Waters et al., 2012). Karrikin and strigolactone signaling can be distinguished by DWARF14 family members KAI2 and AtD14, respectively. Although their physiological function are different, strigolactones and karrikins pathways both converge upon MAX2 (Flematti et al., 2013). Activated AtD14 by strigolactones interacts with the F-box protein D3/MAX2 (D3 is the rice ortholog of *Arabidopsis* MAX2) in the SCF^D3/MAX2^–E2 complex to regulate repression of shoot branching, promotion of senescence and secondary thickening, alteration of root growth, and enhancement of stress tolerance (Li and Tran, 2015). Similar to the action of AtD14, activated KAI2 by karrikins interacts with the F-box protein MAX2 in the SCF^MAX2^–E2 complex, but plays a role in inhibition of hypocotyl elongation, promotion of seed germination, alteration of leaf and root hair growth, and potentially enhancement of stress tolerance (Li and Tran, 2015). As previous reported, the *Atd4-1* mutant showed increased shoot branching common to the *max2* mutant (Waters et al., 2012), while *kai2* seedlings resemble *max2* seedlings in having enlarged epinastic cotyledons (Flematti et al., 2013). These results further suggest that MAX2- and KAI2-mediated cotyledon flattening is strigolactone-independent, but the molecular mechanism still require further research.

### MAX2 Might Functions Downstream of the Phototropins to Mediate Cotyledon Flattening

Given that the mutants *max2-2* and *max2-3* had similar cotyledon phenotypes to the *phot1 phot2* double mutant, we hypothesized that MAX2 may function in phototropin-dependent cotyledon flattening. In support of this, we found that the double mutants *phot1 max2-2* and *phot1 max2-3* exhibited identical epinastic cotyledon flattening to the double mutant *phot1 phot2*. The identical cotyledon flattening in the *max2* single mutants and the *phot1 max2* double mutants, as well as in *phot1 phot2*, suggests that MAX2 may function downstream of phot1 or both phototropins to mediate phototropin-dependent cotyledon flattening.

Phot1 and phot2 are known to redundantly regulate HBL-induced hypocotyl phototropism (Zhao et al., 2013); however, whether MAX2 regulates phototropin-mediated hypocotyl phototropism is yet to be revealed. Here, we found that the mutants *max2-2* and *max2-3* exhibit normal phototropism in response to both high and low levels of blue light, while the double mutants *phot1 max2-2* and *phot1 max2-3* showed impaired phototropism under low blue light, resembling the phenotype of *phot1*. These results indicate that MAX2 activity does not alleviate the disruption of phot1, and from another side proved that MAX2 might functions downstream of the phototropins to mediate cotyledon flattening. Further studies of cotyledon phenotypes of *phot2 max2 and phot1 phot2 max2* will provide persuasive evidence.

### Auxin Distribution May Be Involved in MAX2-Mediated Cotyledon Flattening

The mechanism behind the epinastic cotyledons of the *max2* and *phot1 phot2* mutants was further investigated. The expression of *MAX2* was evaluated to determine its possible function in cotyledon flattening; however, the mutation of *PHOT1*, *PHOT2*, or both phototropins did not seem to affect the expression of *MAX2*. These results indicate that both MAX2 and the phototropins participate in the regulation of cotyledon flattening, but the mechanism is still unknown.

The distribution of auxin plays an important role in leaf morphogenesis (Lumba and McCourt, 2005), and phototropin-mediated auxin redistribution may be responsible for leaf flattening and positioning (Hobbie and Estelle, 1995; Keller and Van Volkenburgh, 1997; Watahiki and Yamamoto, 1997). Activated phot1 directly phosphorylates ABCB19 (ATP-BINDING CASSETTE B19) to inhibit its auxin efflux activity (Christie et al., 2011; Hohm et al., 2014), and causes the relocalization of the auxin efflux carriers, PIN1 and PIN3 (Blakeslee et al., 2004; Ding et al., 2011). Furthermore, the PKSs, signaling components in the phototropin pathways, were reported to control auxin distribution in the leaves (de Carbonnel et al., 2010; Fankhauser and Christie, 2015). *MAX2* was previously reported to regulate auxin transport (Xie et al., 1998; Yu et al., 2009), and a MAX-dependent strigolactone was previously reported to regulate auxin transport (de Jong et al., 2014). This process is dependent on PIN1 activity but independent of AXR1 (AUXIN RESISTANT1)-mediated auxin signaling (Bennett et al., 2006). The further study of auxin transport will help us to better elucidate the mechanisms of MAX2 and phototropin-dependent cotyledon flattening.

## Materials and Methods

### Plant Materials and Growth Conditions

The WT *Arabidopsis thaliana* ecotype used was Col-0, except for the *Landsberg erecta* used for the map-based cloning. The *phot1* (*phot1-5*), *phot2* (*phot2-1*), and *phot1 phot2* (*phot1-5 phot2-1*) lines were gifted by Ken-ichiro Shimazaki, while the *max1* (CS9564), *max2*-2 (SALK_028336C), *max2*-*3* (SALK_092836C), *max3-12* (SALK_015785C), and *max4* (SALK_082552C), lines were purchased from The European Arabidopsis Stock Centre (NASC). The *phot1* seeds were mutagenized with 0.3% (w/v) EMS, as previously described (Zhao et al., 2017).

### Isolation and Identification of *phot1* *lea1* Mutants

The M_2_ seeds were sown on MS medium supplemented with 0.6% (w/v) agar and 3% (w/v) sucrose. After vernalization for 3 days in the dark at 4°C, the seeds were germinated in a phytotron growth cabinet (100 μmol m^−2^ s^−1^ continuous white light, 22°C). After 7 days, the cotyledonary petiole angle and cotyledon angle were measured. Mutants were selected for defects in leaf flattening, and were grown in soil. The seeds of these plants were harvested for genetic and physiological identification.

The mutants were analyzed as described previously (Zhao et al., 2013). Steadily heritable mutants were used as the female parent in a backcross with Col-0 or a cross with *Landsberg erecta* to obtain F_1_ plants, which were self-fertilized to obtain the F_2_ generation. The phenotypic segregation of the F_2_ plants was used to confirm the hereditary character of the mutation.

### Plasmid Construction and Plant Transformation

A genomic DNA fragment containing the entire *MAX2* coding region and the approximately 2.0-kb upstream sequence was amplified using PCR and cloned into a modified pCAMBIA-1300 vector (the 35S CaMV promoter was removed). The plasmids with the correct *MAX2* DNA sequence were introduced into the *phot1 lea1* mutant an Agrobacterium-mediated transformation using the floral dip method. The transgenic plants were selected on a medium containing hygromycin (25 μL mL^−1^), and the successfully transformed plants were used for further phenotypic analysis.

To generate the *MAX2-GUS* construct, the promoter sequence of *MAX2* (2.0-kb upstream sequence of *MAX2*) was amplified using PCR and cloned into the vector pCAMBIA-1391 to construct the recombinant MAX2-GUS plasmid. The plasmids with the correct *MAX2* promoter sequence were introduced into Col-0 in an Agrobacterium-mediated transformation using the floral dip method. The positive transformants were selected on a medium containing hygromycin.

### Measurement of the Cotyledon Angle and the Cotyledonary Petiole Angle

The cotyledon angle and the cotyledonary petiole angle were measured as described previously (Harada et al., 2013). After vernalization for 3 days in the dark at 4°C, seeds were sown on 1.0% MS medium and placed vertically in the growth room with a white fluorescent light irradiated from above. After growth under a 16/8 h light/dark cycle at 22–24°C for about 10 days, images of the seedlings were taken with a Cannon camera. The cotyledon angles, the angle between the petiole and cotyledonary blade, and the cotyledonary petiole angle, the angle between the cotyledonary petiole and the horizontal plane were measured using an e-ruler.

### Histochemical Detection of GUS Activity

The transgenic plants containing the recombinant MAX2-GUS plasmid were analyzed after an incubation in X-gluc buffer (50 mM sodium phosphate buffer (pH 7.0), 10 mM EDTA, 0.1% Triton X-100, 0.5 mM potassium ferrocyanide, and 2 mg mL^−1^ 5-bromo-4-chloro-3-indolyl β-D-glucuronide [X-gluc]) at 37°C for 12 h.

### Measurement of Hypocotyl Curvature and Length

The hypocotyl curvatures were measured as described previously (Zhao et al., 2018). Briefly, etiolated seedlings with hypocotyls 5–8 mm in length were transferred to 0.8% MS medium and placed vertically in a darkroom. After a 12-h irradiation with unilateral blue light (100 μmol m^−2^ s^−1^ or 0.01 μmol m^−2^ s^−1^), the seedlings were photographed using a Canon camera, and their hypocotyl curvatures were measured using an e-ruler. The length of the seedlings was also measured.

## Author Contributions

XoZ and XnZ designed the research, analyzed the data, and contributed reagents and materials. Q-PZ, X-NW, Z-YZ, S-CM, and N-NL performed the experiments. XoZ, XnZ, and Q-PZ wrote the article. All authors have read and approved this manuscript.

## Conflict of Interest Statement

The authors declare that the research was conducted in the absence of any commercial or financial relationships that could be construed as a potential conflict of interest.
