# Supplementary figures and images for: Functional Analysis of MAX2 in Phototropins-Mediated Cotyledon Flattening in Arabidopsis
Source: Front Plant Sci. 2018 Oct 17;9:1507. doi: 10.3389/fpls.2018.01507 (PMC6199895; doi:10.3389/fpls.2018.01507)

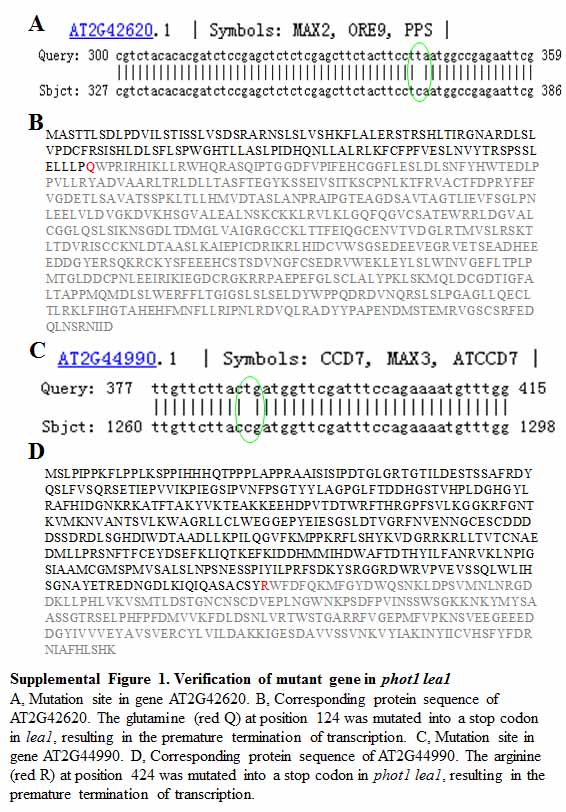

Supplement: Supplementary file 1 [file Image_1.jpg]

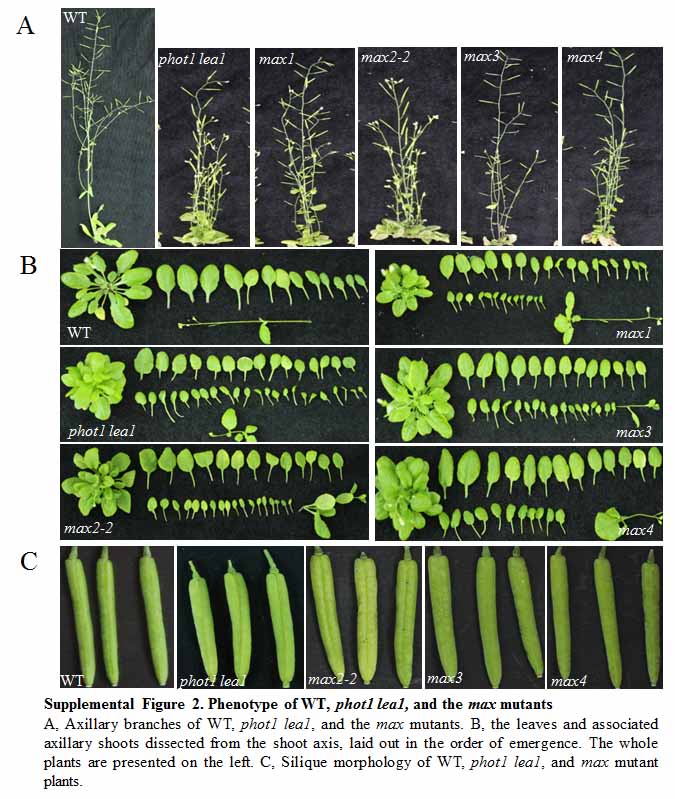

Supplement: Supplementary file 2 [file Image_2.jpg]

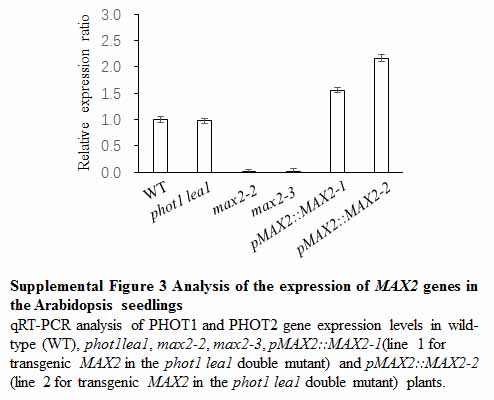

Supplement: Supplementary file 3 [file Image_3.jpg]

**Table 1** Genetic analysis of mutants


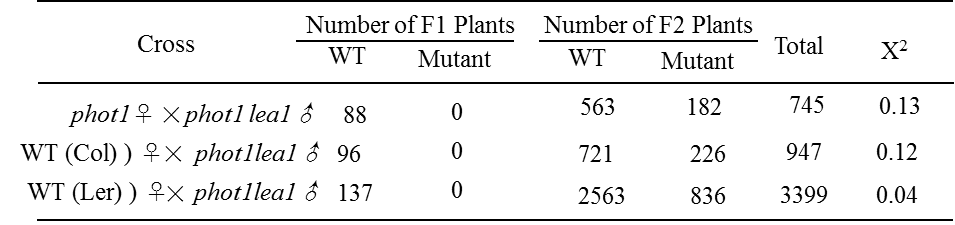

Supplement: Supplementary file 4 [file Table_1.docx]
