# Supplementary material for: Functional Analysis of MAX2 in Phototropins-Mediated Cotyledon Flattening in Arabidopsis
Source: Front Plant Sci. 2018 Oct 17;9:1507. doi: 10.3389/fpls.2018.01507 (PMC6199895; doi:10.3389/fpls.2018.01507)
Supplement: Supplementary file 5 [file Table_2.docx]

**Table 2** Primers and recombinant frequencies for marker locations used for coarse genome mapping

| BAC name | Marker location | Forward primer sequence | Reverse primer sequence | Sample | RF (%) |
| --- | --- | --- | --- | --- | --- |
| T23J18 | Upstream of Chr. 1 | GATATTTGTTTTGCTAACAC | TAATAAAGTTCCAGCTTTG | 96 | 52.60 |
| F28H19 | Middle of Chr. 1 | TGCGGGAGTGTGATAGAAT | TCCTCGAAAGATTCATTGA | 93 | 46.65 |
| T17F3 | Downstream of Chr. 1 | GGACCGACGGTTACGAGAG | TAACGGGCCGTTGCAAGA | 96 | 53.44 |
| T23K3 | Upstream of Chr. 2 | CGTGTTTACCGGGTCGGA | AAAACCCTTGAAGAATAC | 95 | 49.14 |
| F3P11 | Middle of Chr. 2 | ATGTATTTGTTGCAAAATA | TGCACAGAAGAAAAAACT | 94 | 28.44 |
| **T16B24** | Downstream of Chr. 2 | ATGAACGGAGTAGCTATC | CGCGTAGAACATAATCTGT | 93 | 12.93 |
| F20H23 | Upstream f Chr. 3 | CAATGGGAAGAAGGTGTGA | CGCATTTCCATAAGTTTGTT | 92 | 52.63 |
| K1G2 | Middle of Chr. 3 | ATGAGCTTTAGGAGTGTGT | AATTTTGTCCCAAAAGAATA | 93 | 53.44 |
| T26I12 | Downstream of Chr. 3 | GAGCAACATTAAGGATAGA | ATCTCATACTCATAATATGT | 96 | 58.30 |
