# Supplementary material for: Functional Analysis of MAX2 in Phototropins-Mediated Cotyledon Flattening in Arabidopsis
Source: Front Plant Sci. 2018 Oct 17;9:1507. doi: 10.3389/fpls.2018.01507 (PMC6199895; doi:10.3389/fpls.2018.01507)
Supplement: Supplementary file 6 [file Table_3.docx]

**Table 3** Primers and recombinant frequencies for marker locations used for fine genome mapping

| BAC name | Forward primer sequence | Reverse primer sequence | Sample | RF (%) |
| --- | --- | --- | --- | --- |
| T2P4 | AATTCTGGTCAGCAACA | GAGAGGATGGCATAGGT | 641 | 5.9 |
| F13H10 | TGTGTGAATCGTGATTGA | CACCACCATGTAAAACTTAG | 625 | 1.20 |
| T6D20 | TGTTTGCATTTGATGTTC | AAATGTCATAAATTAAGAGA | 623 | 0.98 |
| **F14N22** | CAATACTAGACGTCTTAAATGG | CATAGATAAGCTGTCGTTAATC | 643 | 0.56 |
| T1O24 | GACTCATTGATTACGCTAGG | TCATCACCACATTGGTAGTT | 635 | 1.23 |
| F6E13 | CAATTGTGTGGTGAATGAA | TGGGCATCTCTCTGGGTA | 637 | 1.79 |
| F16B22 | CGTGAATGCTTTAGGAGTCC | TGGATTCGTTGCATGATGCG | 827 | 3.45 |
