# Supplementary material for: Functional Analysis of MAX2 in Phototropins-Mediated Cotyledon Flattening in Arabidopsis
Source: Front Plant Sci. 2018 Oct 17;9:1507. doi: 10.3389/fpls.2018.01507 (PMC6199895; doi:10.3389/fpls.2018.01507)
Supplement: Supplementary file 7 [file Table_4.docx]

**Table 4** Descriptions of predicted candidate genes

| Gene name | Description of protein |
| --- | --- |
| AT2G42500 | One of the isoforms of the catalytic subunit of protein phosphatase 2A |
| AT2G42510 | Spliceosome-related protein involved in spliceosome assembly and nuclear mRNA splicing |
| AT2G42520 | P-loop-containing nucleoside triphosphate hydrolase superfamily protein, which functions in helicase activity, nucleic acid binding, ATP binding, and ATP-dependent helicase activity |
| AT2G42530 | COR15B, which protects the chloroplast membrane during freezing |
| AT2G42540 | A cold-regulated gene whose product is targeted to the chloroplast |
| AT2G42550 | Protein kinase superfamily protein involved in protein amino acid phosphorylation |
| AT2G42560 | Late embryogenesis abundant group 4 protein, |
| AT2G42570 | A member of the TBL (TRICHOME BIREFRINGENCE-LIKE) gene family containing a plant-specific DUF231 (domain of unknown function) domain |
| AT2G42580 | Member of the TTL family required for osmotic stress tolerance and male sporogenesis |
| AT2G42590 | A 14-3-3 protein which functions in binding calcium and displays induced structural changes |
| AT2G42600 | One of four Arabidopsis phosphoenolpyruvate carboxylase proteins |
| AT2G42610 | Protein of unknown function |
| **AT2G42620** | A member of the F-box leucine-rich repeat family of proteins involved in leaf senescence, regulation of light responses, inhibition of hypocotyl, and petiole elongation in light-grown seedlings |
| AT2G42640 | Mitogen activated protein kinase kinase kinase-related protein involved in amino acid phosphorylation |
| AT2G42650 | Ribosomal protein L1p/L10e family member, functions in RNA binding |
| AT2G42660 | Myb-like HTH transcriptional regulator family protein |
| AT2G42670 | Protein of unknown function |
| AT2G42680 | Bridging factor between a bZIP factor and TBP |
| AT2G42690 | Alpha/beta-hydrolase superfamily protein with triglyceride lipase activity, involved in lipid metabolic process |
| AT2G42700 | Sec1-like protein involved in vesicle-mediated transport and vesicle docking in exocytosis |
| AT2G42710 | Structural constituent of ribosome involved in translation, RNA processing, and binding |
| AT2G42720 | Protein contains FBD, F-box, Skp2-like, and leucine-rich repeat domains |
